# Supplementary material for: Comprehensive characterisation of IAA inactivation pathways reveals the impact of glycosylation on auxin metabolism and plant development in Arabidopsis
Source: Commun Biol. 2026 Jun 4;9:762. doi: 10.1038/s42003-026-10431-5 (PMC13237164; doi:10.1038/s42003-026-10431-5)
Supplement: Supplementary file 1 — Supplementary Information [file 42003_2026_10431_MOESM1_ESM.pdf]

Comprehensive characterisation of IAA inactivation pathways reveals the impact of glycosylation on auxin metabolism and plant development in Arabidopsis

Rubén Casanova-Sáez, Aleš Pěnčík, Federica Brunoni, Anita Ament, Pavel Hladík, Asta Žukauskaitė, Jan Šimura, Ute Voß, Ondřej Novák, Malcolm Bennett, Karin Ljung, and Eduardo Mateo-Bonmatí

Supplementary Information

Supplementary Figures

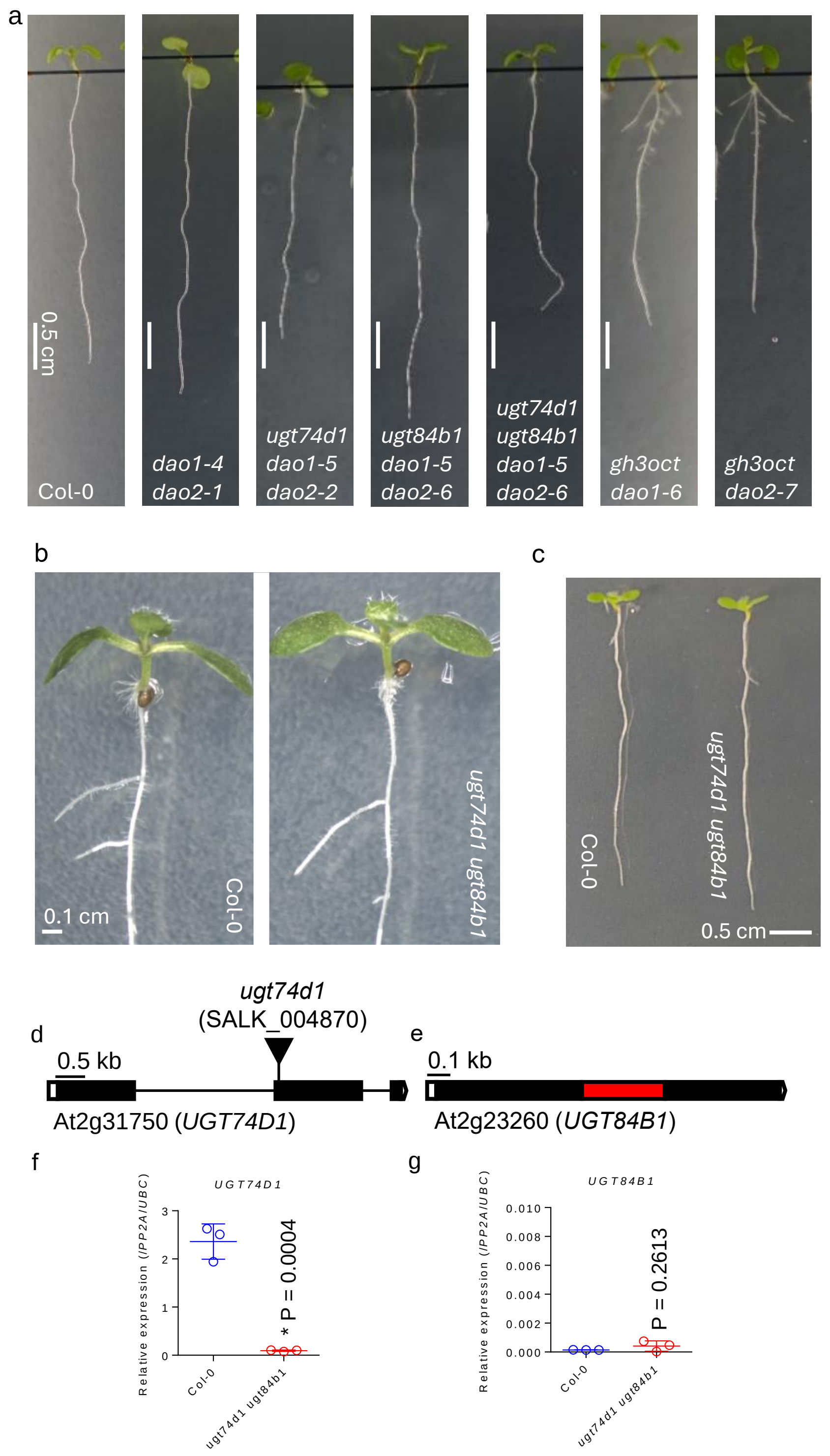

Figure S1. Phenotype and characterization of mutants involved in IAA inactivation.(a) Root phenotype of the assorted mutants grown vertically for 7 days. (b, c) Details of the hypocotyl and root phenotype of *ugt74d1 ugt84b1*. (d, e) Gene structure indicating the mutations studied. The triangle represents a T-DNA insertion. The red rectangle indicates a CRISPR/Cas9-induced deletion. (f, g) Relative expression levels of *UGT74D1* and *UGT84B1* in the *ugt74d1 ugt84b1* mutant. Expression values were normalized to the *PP2A* and *UBC* housekeeping genes. As previously shown in Mateo-Bonmatí et al. (2021), *UGT74D1* levels are significantly reduced compared to the wild type ($p < 0.05$; Student's t-test), whereas *UGT84B1* expression levels are not affected by the CRISPR/Cas9 editing. Scale bars represent: (a, c) 0.5 cm; (b) 0.1 cm.

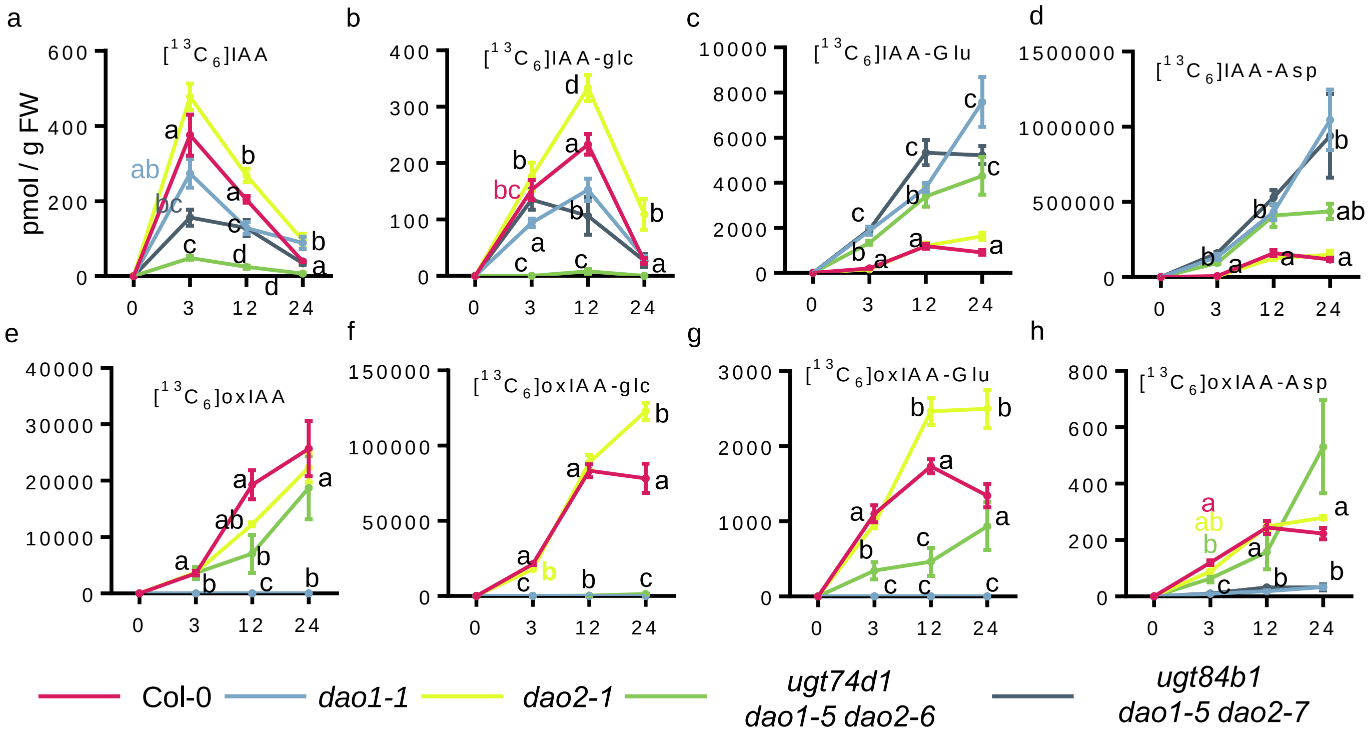

Figure S2. Levels of *de novo* synthesized indole-3-acetic acid (IAA) metabolites in assorted genotypes. (a–h) Formation of [$^{13}C_6$]-labelled IAA metabolites in 7-day-old seedlings of the indicated genotypes after incubation with 1 μM [$^{13}C_6$]IAA for 0, 3, 12, and 24 h. Dots indicate the mean ± standard error of the mean. Levels, expressed as picomoles per gram fresh weight, of (a) [$^{13}C_6$]IAA, (b) [$^{13}C_6$]IAA-glc, (c) [$^{13}C_6$]IAA-Glu, (d) [$^{13}C_6$]IAA-Asp, (e) [$^{13}C_6$]oxIAA, (f) [$^{13}C_6$]oxIAA-glc, (g) [$^{13}C_6$]oxIAA-Glu, and (h) [13C6]oxIAA-Asp. For each time point, differences were evaluated by one-way ANOVA followed by pairwise comparisons using Tukey's HSD test.

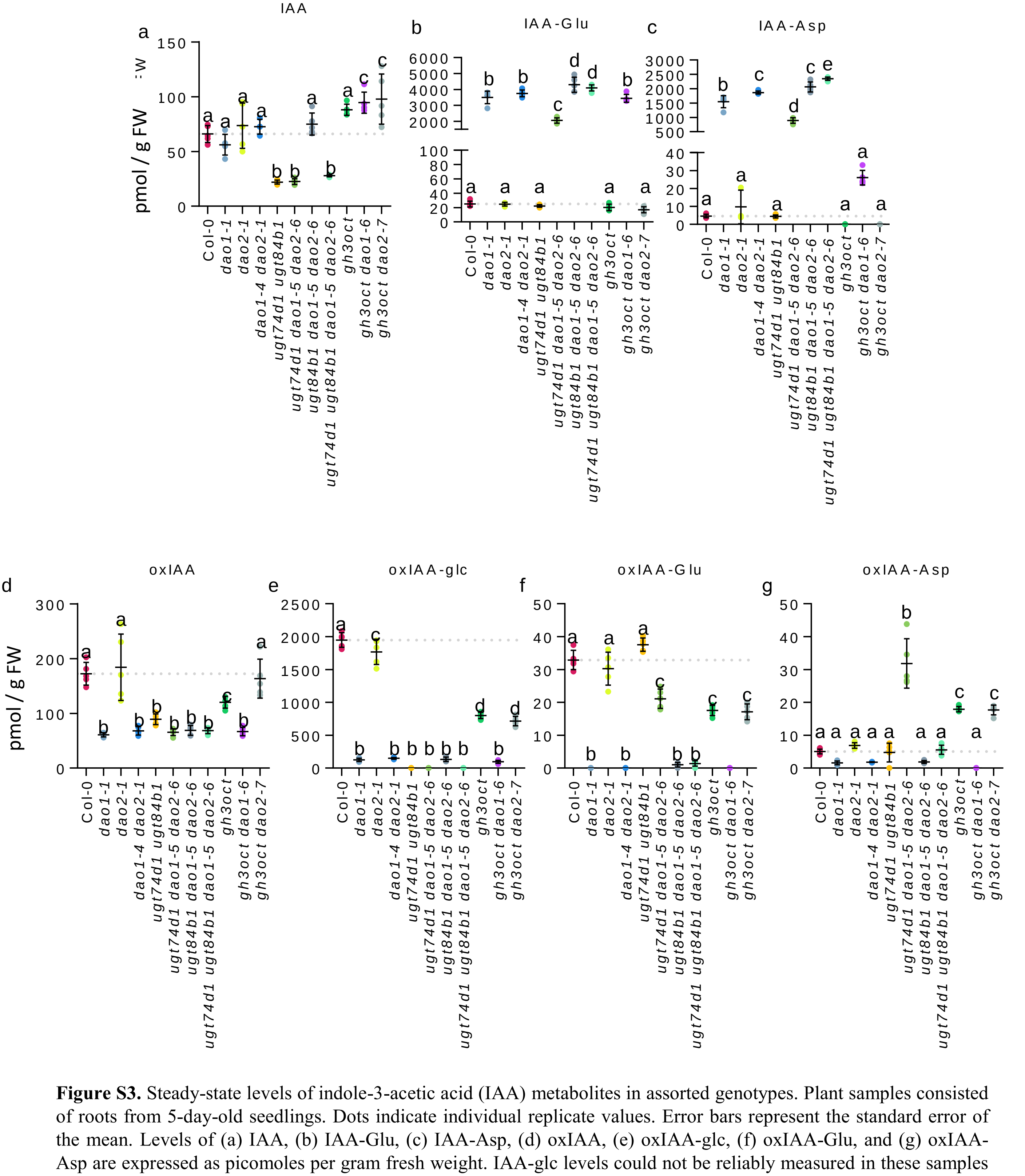

Figure S3. Steady-state levels of indole-3-acetic acid (IAA) metabolites in assorted genotypes. Plant samples consisted of roots from 5-day-old seedlings. Dots indicate individual replicate values. Error bars represent the standard error of the mean. Levels of (a) IAA, (b) IAA-Glu, (c) IAA-Asp, (d) oxIAA, (e) oxIAA-glc, (f) oxIAA-Glu, and (g) oxIAA-Asp are expressed as picomoles per gram fresh weight. IAA-glc levels could not be reliably measured in these samples due to an unknown co-eluting compound masking detection of the IAA-glc peak. Differences were evaluated by one-way ANOVA followed by pairwise comparisons using Tukey's HSD test**.**

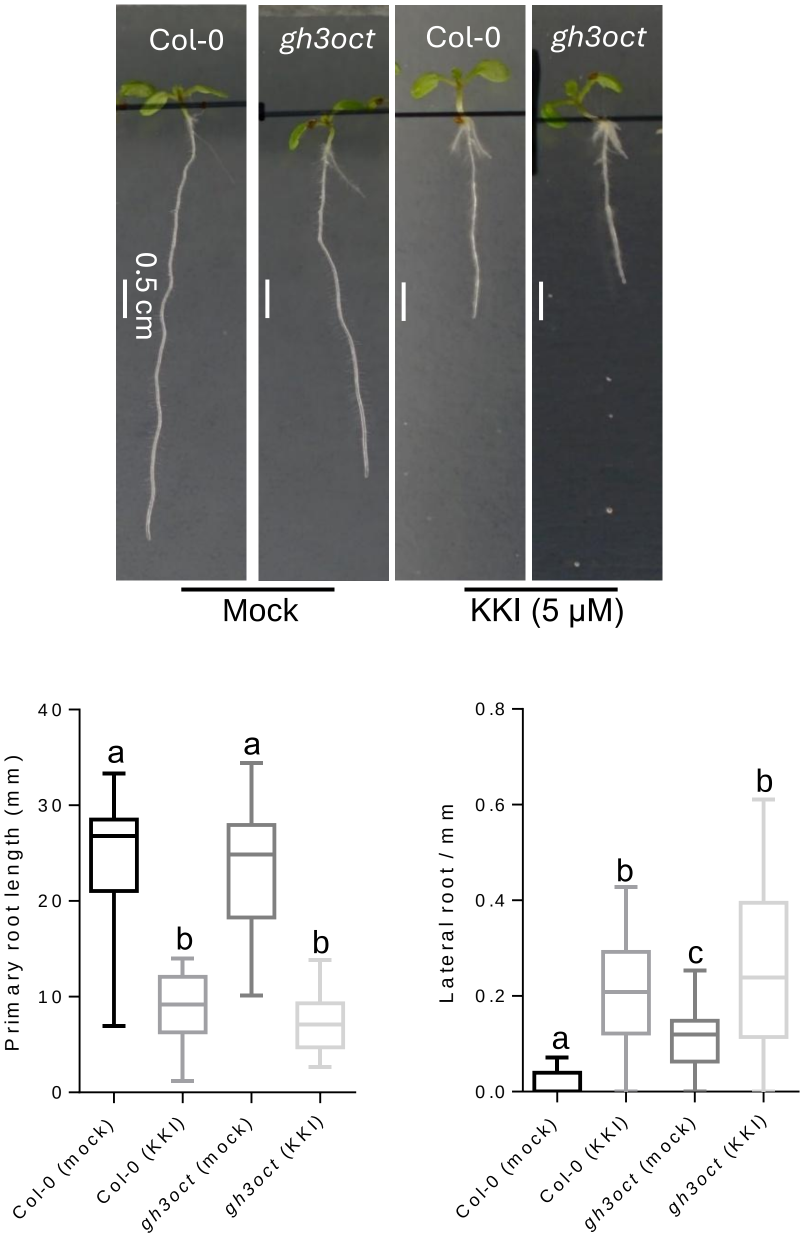

Figure S4. Morphological traits of Col-0 and *gh3oct* in response to kakeimide. Representative images of Col-0 and *gh3oct* grown under mock conditions and in the presence of 5 µM kakeimide (KKI). Primary root length and lateral root density were measured in the indicated genotypes and treatments. Scale bars represent 0.5 cm. Differences were evaluated by two-way ANOVA followed by pairwise comparisons using Tukey's HSD test (n > 23).

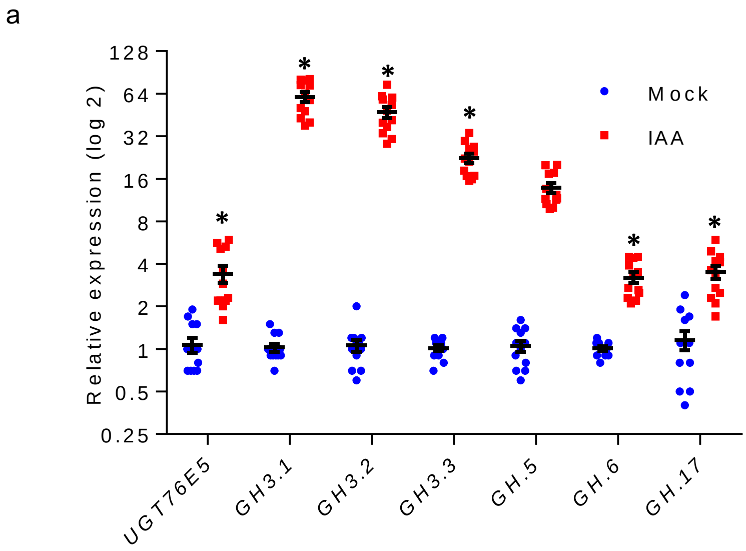

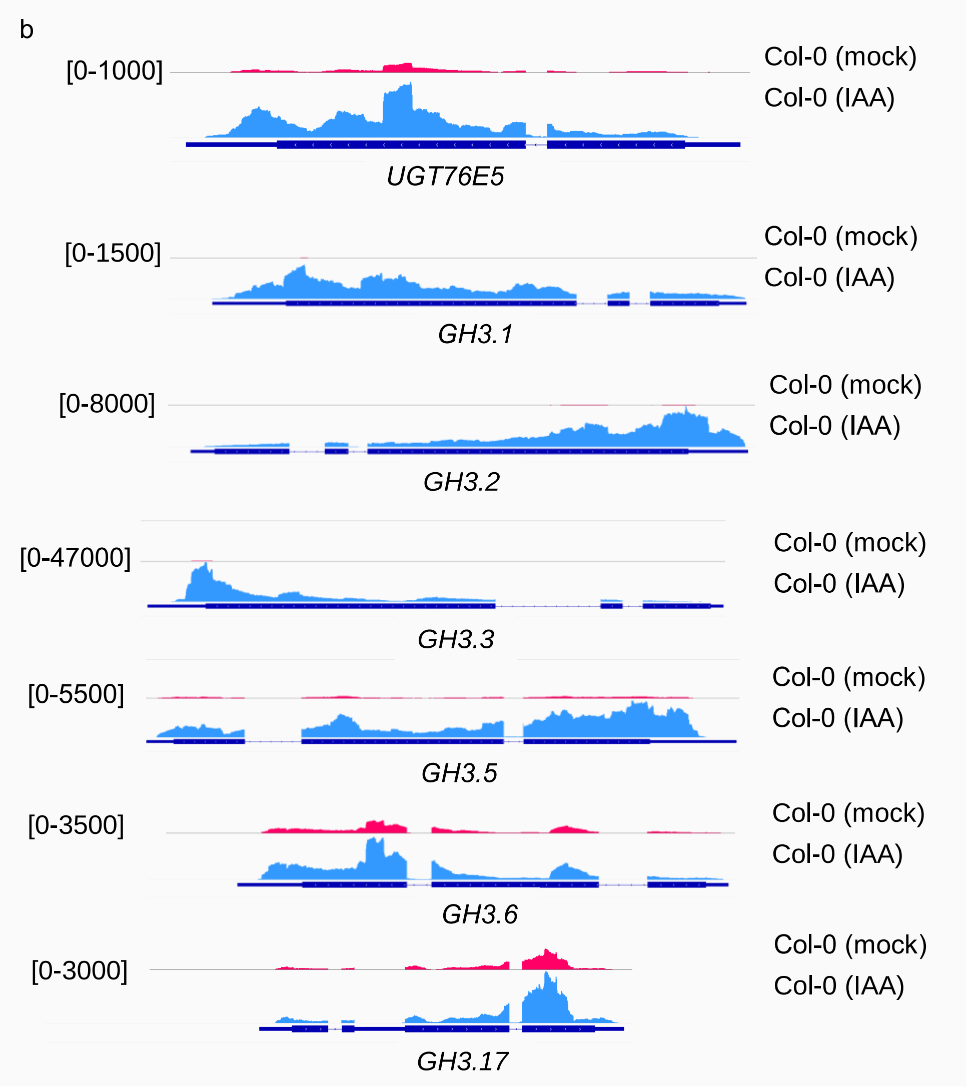

Figure S5. Assessment of gene induction under the conditions used for the RNA-seq analysis. (a) Relative expression levels of the IAA-responsive genes *UGT76E5*, *GH3.1*, *GH3.2*, *GH3.3*, *GH3.5*, *GH3.6*, and *GH3.17* from Col-0 plants grown for five days on a nylon mesh and transferred to a plate containing 1 µM IAA for 4 hours. Values were normalized to the mock treatment. An asterisk indicates statistically significant differences relative to mock treatment (* $p < 0.0001$; n = 12; Mann-Witney *U* test). (b) Screenshots of RNA-seq profiles for the same genes shown in (a) from the transcriptomics experiment. Profiles were visualized in IGV and scaled to the signal in IAA-treated samples. The upper and lower panels correspond to Col-0 mock and IAA-treated, respectively.

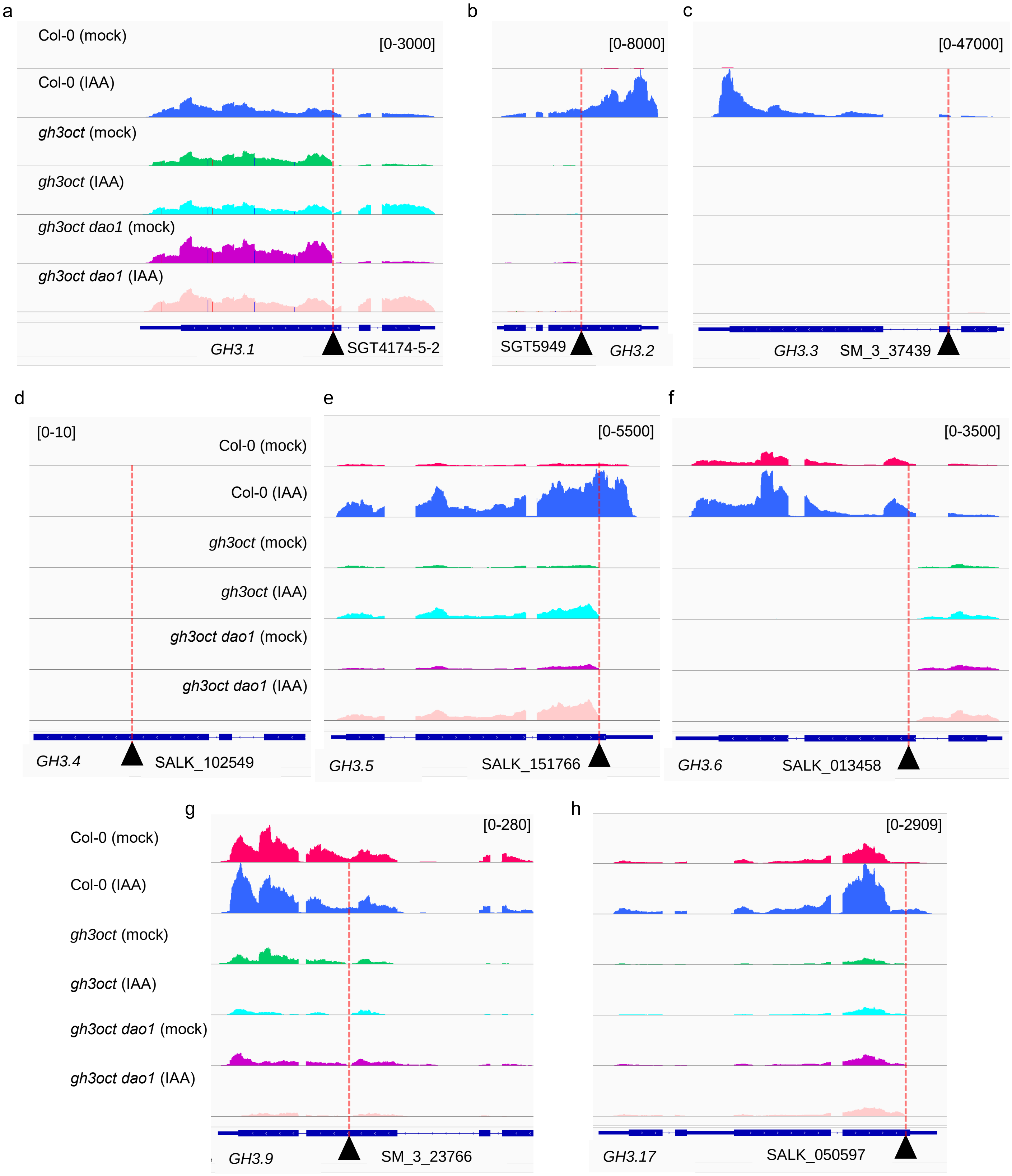

Figure S6. Analysis of T-DNA insertions in *gh3oct*. (a–h) IGV tracks of Col-0 (mock), Col-0 (IAA-treated), *gh3oct* (mock), *gh3oct* (IAA-treated), and *gh3oct dao1-6* (mock and IAA-treated), shown for (a) *GH3.1*, (b) *GH3.2*, (c) *GH3.3*, (d) *GH3.4*, (e) *GH3.5*, (f) *GH3.6*, (g) *GH3.9*, and (h) *GH3.17*. The bottom track corresponds to gene annotation. Triangles indicate the insertion sites in each gene. A red dashed line marks the insertion point. Coloured vertical lines in panel (a) correspond to reads carrying polymorphisms between Col-0 (reference genome) and L*er* (the original background of some insertional alleles).

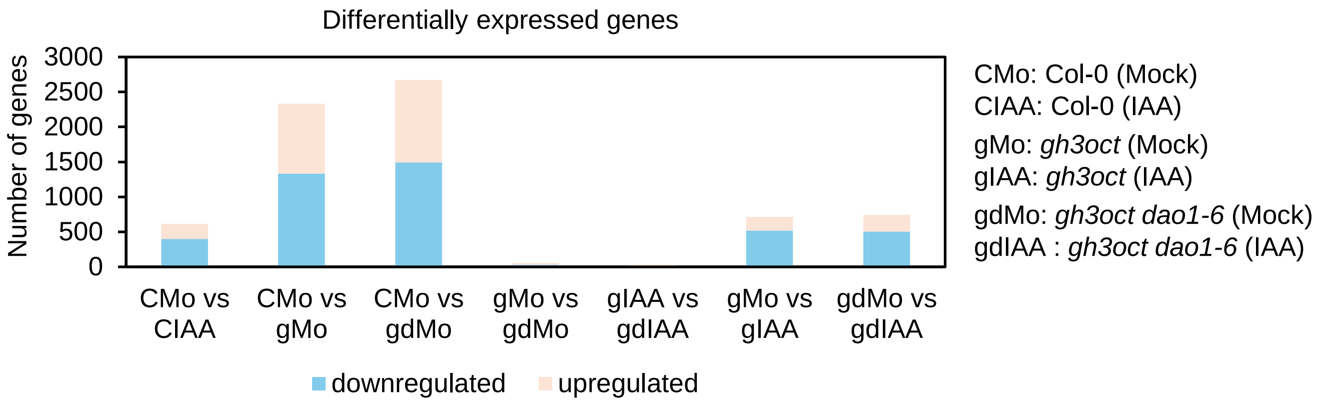

Figure S7. Absolute number of differentially expressed genes in the different comparisons performed by RNA-seq.

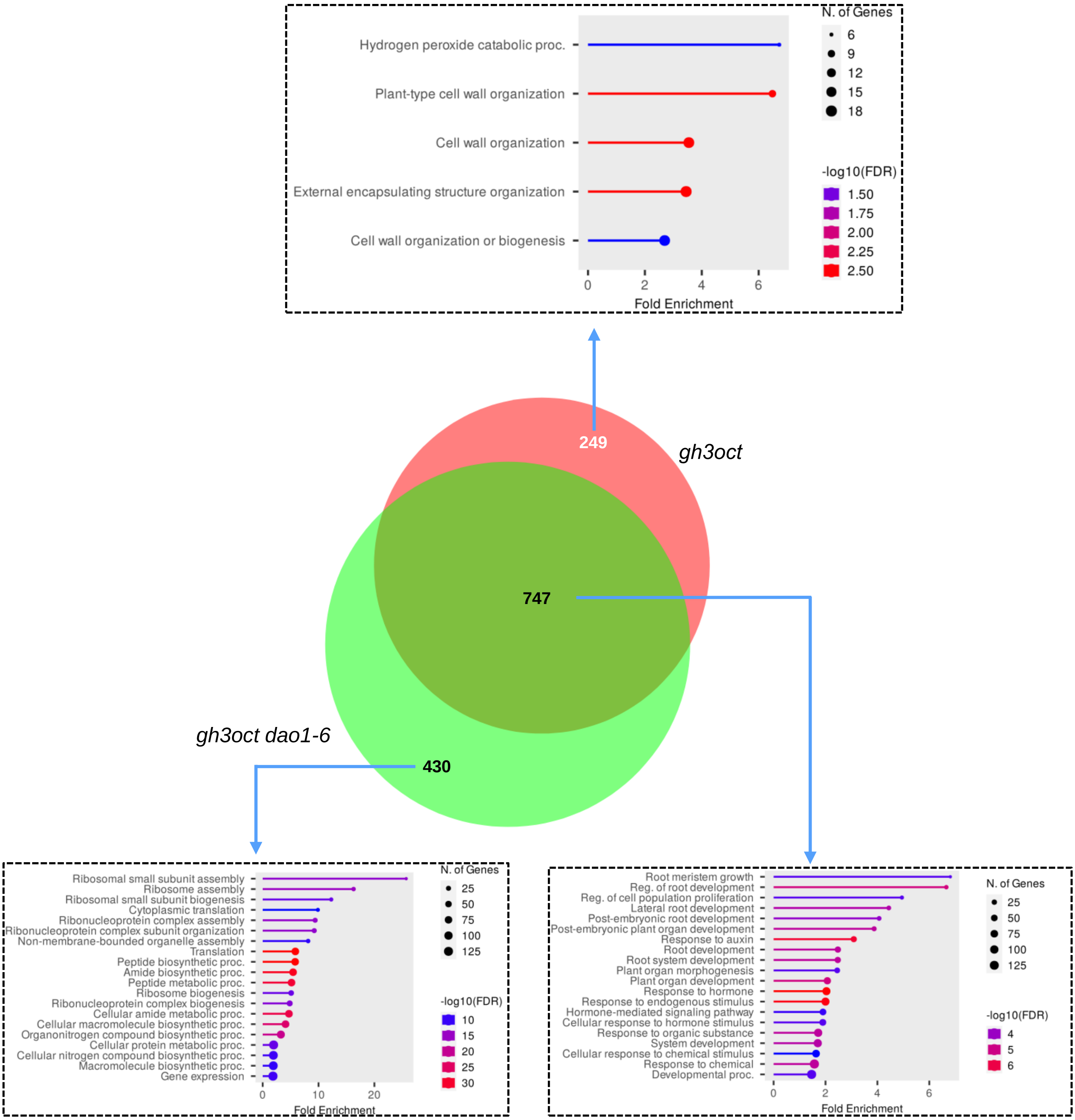

Figure S8. Venn diagram showing the overlap between upregulated genes in *gh3oct* and *gh3oct dao1* under mock conditions. Overrepresented Gene Ontology (GO) terms in the *Biological Process* category are shown as lollipop plots. Lollipop plots were generated using ShinyGO with default parameters. The colour scale corresponds to the −log10 of the false discovery rate (FDR); the size of each lollipop represents the number of genes in each category; and the x-axis indicates the fold enrichment of each category.

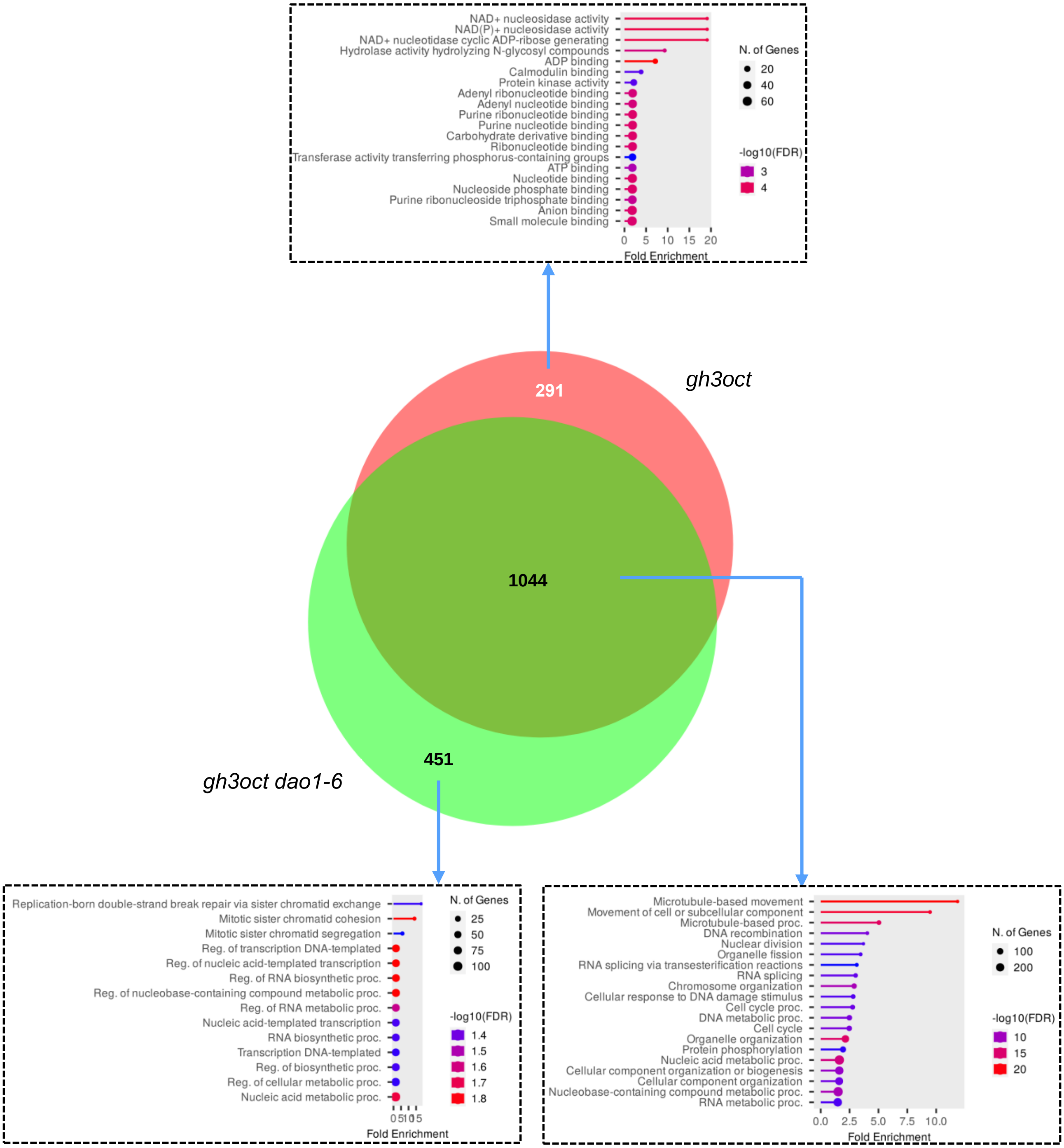

Figure S9. Venn diagram showing the overlap between downregulated genes in *gh3oct* and *gh3oct dao1* under mock conditions. Overrepresented Gene Ontology (GO) terms in the *Biological Process* (bottom panels) and *Molecular Function* (top panel) categories are shown as lollipop plots. Lollipop plots were generated using ShinyGO with default parameters. The colour scale corresponds to the −log10 of the false discovery rate (FDR); the size of each lollipop represents the number of genes in each category; and the x-axis indicates the fold enrichment of each category.

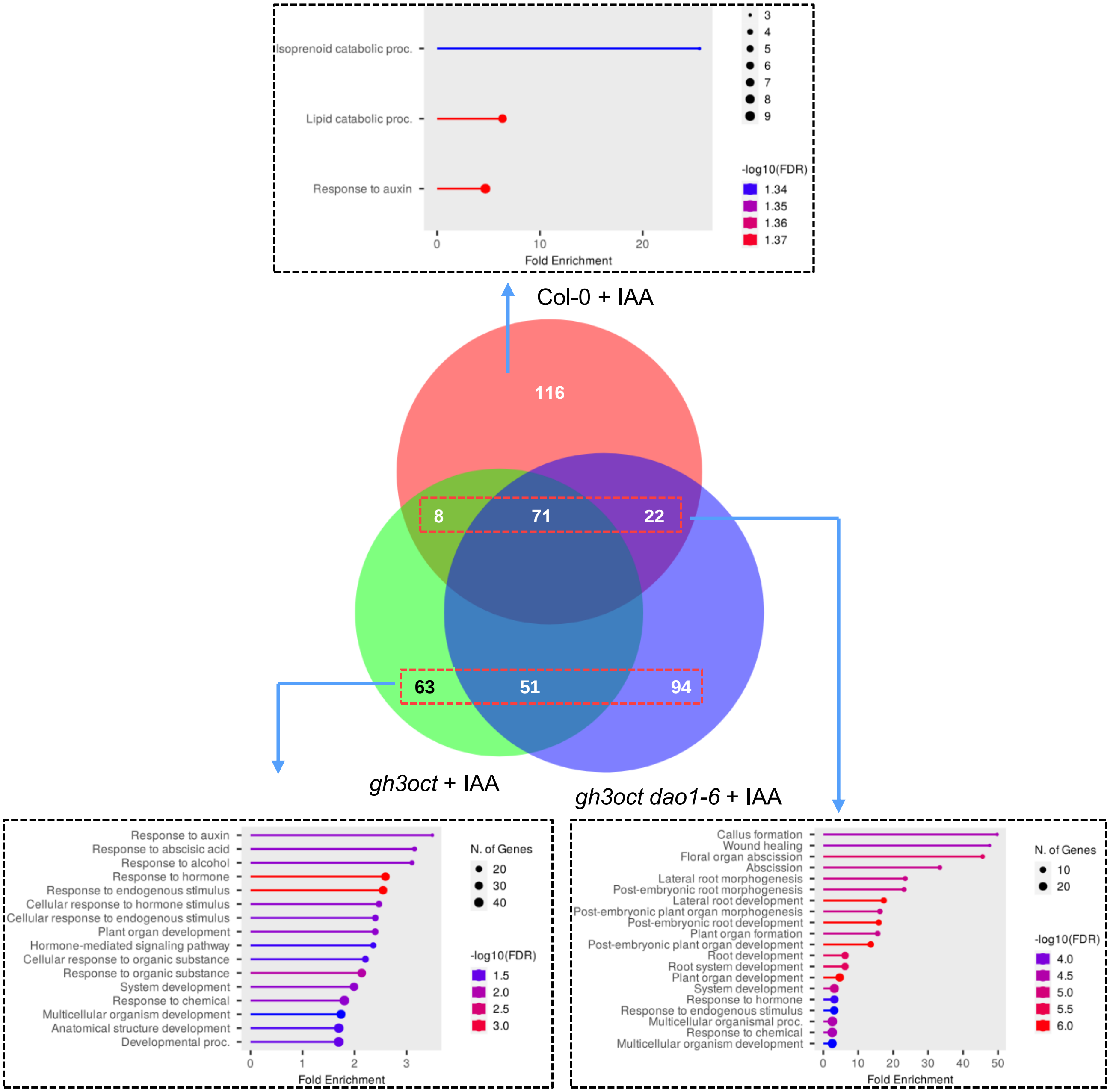

Figure S10. Venn diagram showing the overlap between genes upregulated upon IAA treatment in Col-0, *gh3oct*, and *gh3oct dao1*. Overrepresented Gene Ontology (GO) terms in the *Biological Process* category are shown as lollipop plots. Lollipop plots were generated using ShinyGO with default parameters. The colour scale corresponds to the −log10 of the false discovery rate (FDR); the size of each lollipop represents the number of genes in each category; and the x-axis indicates the fold enrichment of each category.

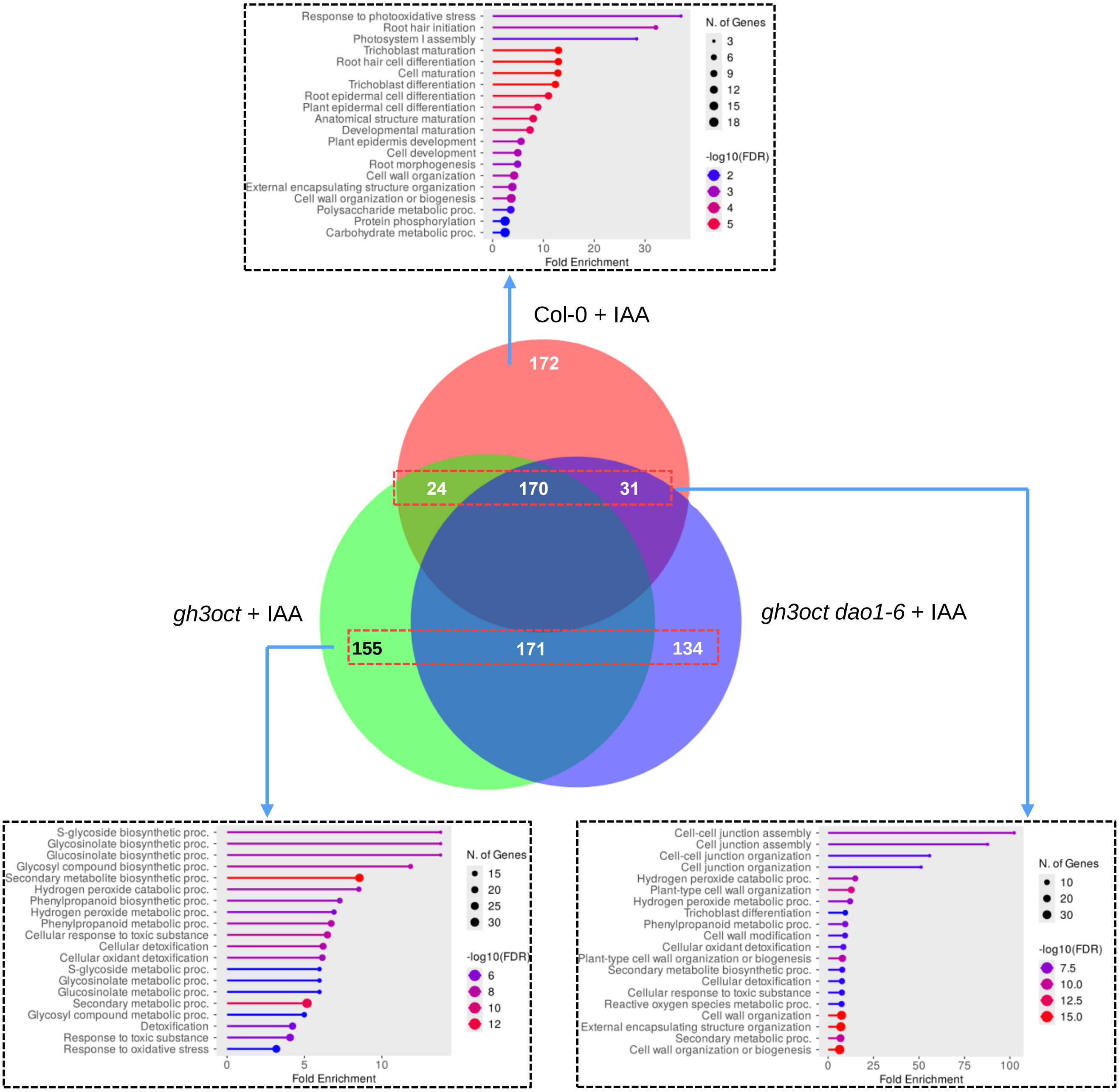

Figure S11. Venn diagram showing the overlap between genes downregulated upon IAA treatment in Col-0, *gh3oct*, and *gh3oct dao1*. Overrepresented Gene Ontology (GO) terms in the *Biological Process* category are shown as lollipop plots. Lollipop plots were generated using ShinyGO with default parameters. The colour scale corresponds to the −log10 of the false discovery rate (FDR); the size of each lollipop represents the number of genes in each category; and the x-axis indicates the fold enrichment of each category.

Casanova-Sáez *et al.* Figure S12

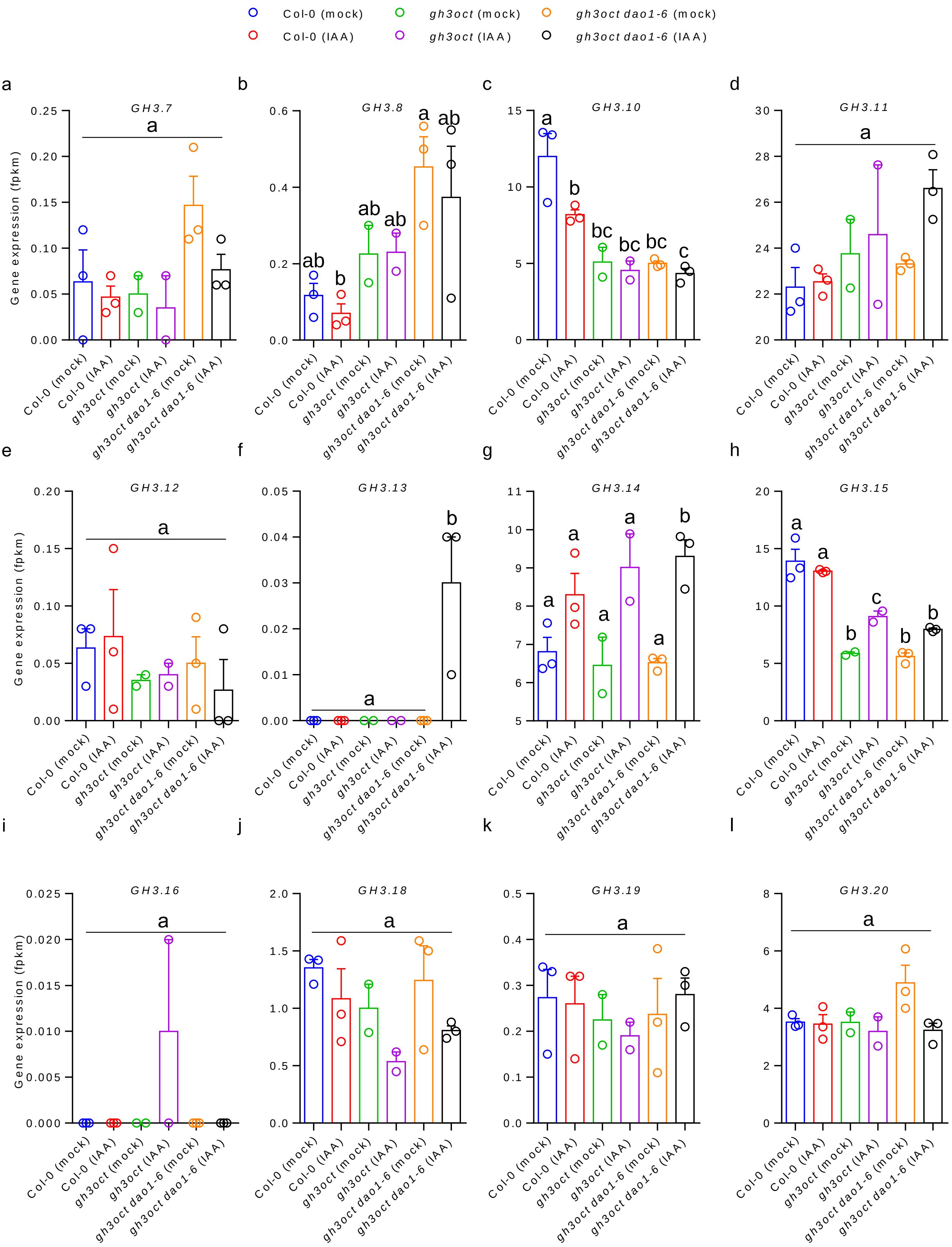

Figure S12. Differences in expression of non-group II *GH3* genes. Expression levels are reported as fragments per kilobase of transcript per million reads mapped (FPKM). Error bars indicate the standard error of the mean. For each genotype, mock (left) and IAA (right) treatments are shown. Differences were evaluated by two-way ANOVA followed by pairwise comparisons using Tukey's HSD test.

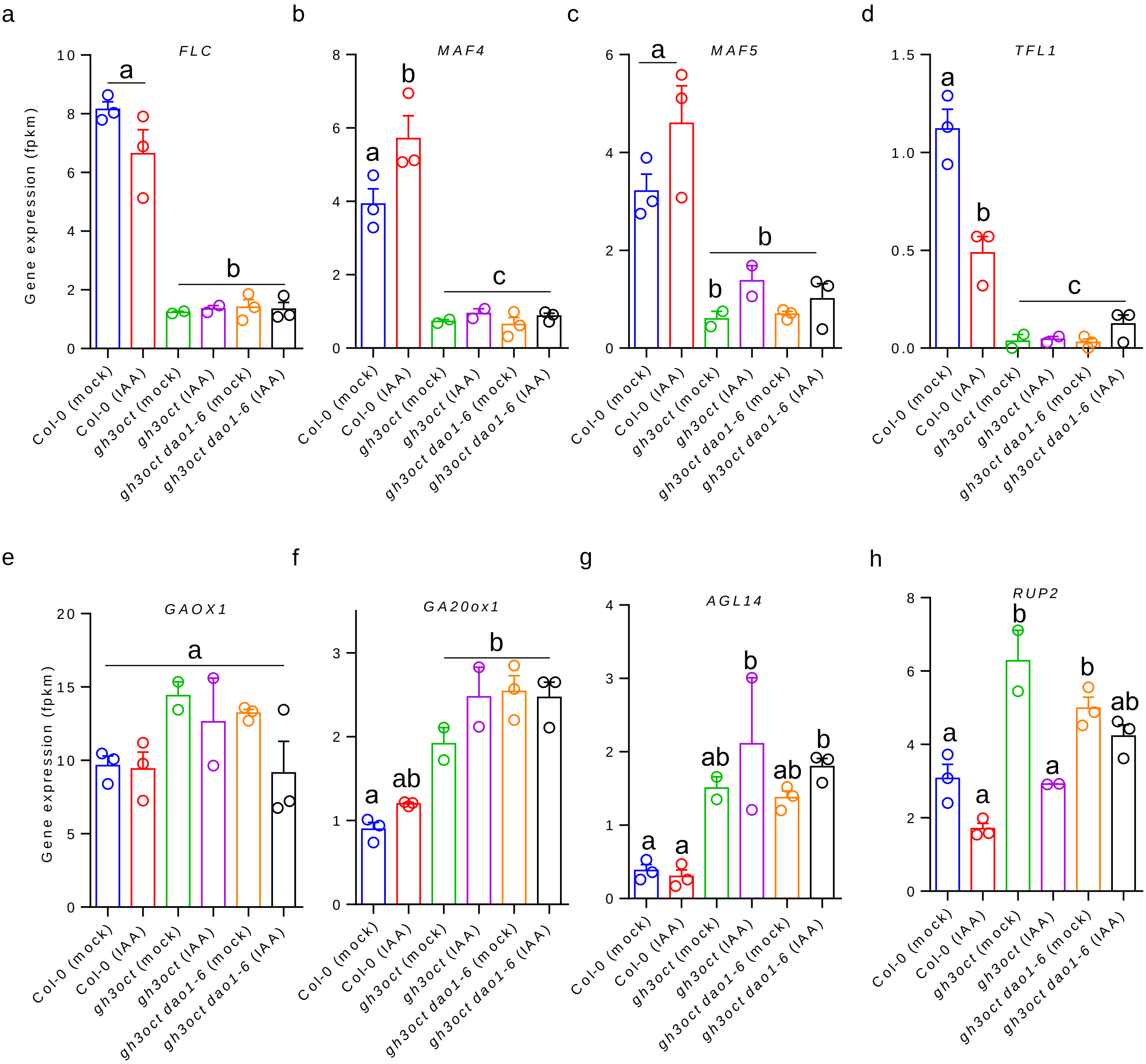

Figure S13. Differences in expression of flowering time-related genes. Expression levels of flowering (a–d) repressor and (e–h) activator genes are reported as fragments per kilobase of transcript per million reads mapped (FPKM). Error bars indicate the standard error of the mean. For each genotype, mock (left) and IAA (right) treatments are shown. Differences were evaluated by two-way ANOVA followed by pairwise comparisons using Tukey's HSD test.

Supplementary Tables

Supplementary Table 1. Primer sets used in this work

Purpose	Primer name	Primer sequence (5' → 3')	
		Forward sequence (F)	Reverse sequence (R)
CRISPR/ Cas9 cloning	DAO1_g#1	AATGTCTCTTTCAACGTCCATACG	AAACCGTATGGACGTTGAAAGAGA
	DAO1_g#2	gTACGCACTTTCACCTCGTAgttttagagctatgctg	TACGAGGTGAAAGTGCGTAcaatcactacttcgactc
	DAO1_g#3	GAGCTCTGTAACCACTCCCTgttttagagctatgctg	AGGGAGTGGTTACAGAGCTCaatcactacttcgactc
	DAO1_g#4	gACGCTTGCTATCAACCTCGgttttagagctatgctg	CGAGGTTGATAGCAAGCGTcaatcactacttcgactc
	DAO1_g#5	gGTACCCCTTCGTGACTAATgttttagagctatgctg	ATTAGTCACGAAGGGGTACcaatcactacttcgactc
	DAO2_g#6	gCCGATGTGTTACTAGGGAAgttttagagctatgctg	TTCCCTAGTAACACATCGGcaatcactacttcgactc
	DAO2_g#7	gTTAGCGGAGAGCTACGGAGgttttagagctatgctg	CTCCGTAGCTCTCCGCTAAcaatcactacttcgactc
Genotyping	UBQ10pro	GTGATCAAGGTAAATTTCTGTGTT	TGAGAAATTGAAATCTGAATTGTG
	B-module	ATGTGAGTTAGCTCACTCATTAG	CAACTGTTGGGAAGGGCGAT
	G-10332	CTCGTATGTTGTGTGGAATTGTGAGC	
	G-37561	TCAGACCTAGAAAAGCTGCAAA	
	G-23253	CCAAACGTAAAACGGCTTGT	
	G-40025	TTGGTCTTGAACAAGAGATC	
	1.2en1.1p-F	AAGCTGATTTGGTTCTATTGAACTA	
	G-33037	GTGTGCGCAATGAAACTGATGC	
	G-37561	TCAGACCTAGAAAAGCTGCAAA	
	mCherry-R1		GGTGCCGCGCAGCTTCACCT
	mCherry-R2		CCTCGCCCTTGCTCACCAT
	Cas9	ATGGATAAGAAGTACTCTATCGG	AACCTTCCTCTTCTTCTTAGGAT
	dao1-5 dao2-6 (wt 3021 / mut 461)	CGGAGTCATCATTCCGACTA	GGCTACGgtgggatgatatc
RT-qPCR	qGH3.1	GGACAACTCGGTTGGACCAT	TGCACCTCTTGAGATTGCGT
	qGH3.2	TGCGTGAGCTTCACACCTAT	CTAAAACCGCACATCATCCG
	qGH3.3	CTCCGTGCCATTGGATTCCT	ATCAGCCAGTTCTTGGTCCG
	qGH3.4	TGATCGGTGTGAGGCTTACG	TGGAGATACGTGTGGTGCAG
	qGH3.5	TGCTCCAATTATCGAGCTATTGA	TGTTTGTGACCAGGAACCCA
	qGH3.6	ACCTATGCTGGGCTTTACAGG	GCGGCATATGAAGCTGAACTG
	qGH3.9	CGACGATGAACAAGTCCCCT	GGTAACGGTACAATCCTGCGA
	qGH3.17	CATTTGTTAAGTTGCTAATTGGTGT	AGAGGACTTTGCTGAAAGTTTGT
	qUGT74D1	TCTCTAAAAACGTCAACGTCACA	CGGAGGATGGAGTTGTGG
	qUGT84B1	TGTAGGTTCTTTGATGATAACGA	CACATGTCCTAATGGTAACAC
	qUBC	CTGCGACTCAGGGAATCTTCTAA	TTGTGCCATTGAATTGAACCC
	qPP2A	ACTGCATCTAAAGACAGAGTTCC	CCAAGCATGGCCGTATCATGT
	qACT2	CCGCTCTTTCTTTCCAAGC	CCGGTACCATTGTCACACAC

Sample	Q20 %	Clean read number	SRA reference
Col-0(Mock)_1	97.66	87,755,182	SAMN16288319
Col-0(Mock)_2	97.53	85,625,344	SAMN16288320
Col-0(Mock)_3	97.47	85,531,692	SAMN16288321
Col-0(IAA)_1	97.61	87,287,732	SAMN16288322
Col-0(IAA)_2	97.73	87,578,238	SAMN16288323
Col-0(IAA)_3	97.69	83,689,142	SAMN16288324
gh3oct(Mock)_1	97.38	87,368,762	SAMN16288325
gh3oct(Mock)_2	97.52	83,285,346	SAMN16288326
gh3oct(IAA)_1	97.72	87,589,734	SAMN16288327
gh3oct(IAA)_2	97.94	87,211,028	SAMN16288328
gh3octdao1(Mock)_1	97.74	87,755,182	SAMN16288329
gh3octdao1(Mock)_2	97.51	86,567,062	SAMN16288330
gh3octdao1(Mock)_3	97.43	87,126,212	SAMN16288331
gh3octdao1(IAA)_1	97.44	87,145,640	SAMN16288332
gh3octdao1(IAA)_2	98.40	84,716,878	SAMN16288333
gh3octdao1(IAA)_3	98.00	81,573,998	SAMN16288334

Supplementary Table 2. Sequencing statistics of the different RNA-seq samples. All the experiments were performed using paired-end (PE) reads of 100 bp in length. Q20 % values indicate the percentage of reads (forward, left; reverse, right) that reach a Phread score of 20 or higher.

Supplementary Table 3. Modules used for the GreenGate-based cloning of the CRISPR-Cas9 plasmids generated in this work.

Construct	Supermodule	Modules	Insert
#1	M	A	EC1.2enhancer–EC1.1promoter
		B	*A.thaliana* codon-optimized Cas9
		C	rbcS terminator
		D	DAO2_g#7
		E	DAO1_g#4
		FH	F-H adapter (pGGG001)
	N	HA	H-A adapter (pGGG002)
		A	UBQ10 promoter
		B	mCherry CDS
		C	rbcS terminator
		D	DAO2_g#6
		E	DAO1_g#3
		F	HygR (pGGF005)
#2	-	A	EC1.2enhancer–EC1.1promoter
		B	*A.thaliana* codon-optimized Cas9
		C	rbcS terminator
		D	DAO1_g#2
		E	DAO1_g#5
		F	HygR (pGGF005)
