# Supplementary material for: Comprehensive characterisation of IAA inactivation pathways reveals the impact of glycosylation on auxin metabolism and plant development in Arabidopsis
Source: Commun Biol. 2026 Jun 4;9:762. doi: 10.1038/s42003-026-10431-5 (PMC13237164; doi:10.1038/s42003-026-10431-5)
Supplement: Supplementary file 2 — Description of Additional Supplementary Files [file 42003_2026_10431_MOESM2_ESM.pdf]

Description of Additional Supplementary Files

File name: Supplementary Data 1

Description: List of IAA-related genes, and lists of genes forming the clusters shown in Figure 7D with indication of the presence of the auxin response cis element in their corresponding.

File name: Supplementary Data 2

Description: List of flowering time-related genes.

File name: Supplementary Data 3

Description: The source data behind the graphs in the paper.
