# Supplementary material for: Comprehensive characterisation of IAA inactivation pathways reveals the impact of glycosylation on auxin metabolism and plant development in Arabidopsis
Source: Commun Biol. 2026 Jun 4;9:762. doi: 10.1038/s42003-026-10431-5 (PMC13237164; doi:10.1038/s42003-026-10431-5)
Supplement: Supplementary file 6 — Reporting Summary [file 42003_2026_10431_MOESM6_ESM.pdf]

nature portfolio

Corresponding author(s): Karin Ljung
Eduardo Mateo-Bonmatí

Last updated by author(s): 19/05/2026

Reporting Summary

Nature Portfolio wishes to improve the reproducibility of the work that we publish. This form provides structure for consistency and transparency in reporting. For further information on Nature Portfolio policies, see our Editorial Policies and the Editorial Policy Checklist.

Statistics

For all statistical analyses, confirm that the following items are present in the figure legend, table legend, main text, or Methods section.

n/a	Confirmed	
☐	☒	The exact sample size (*n*) for each experimental group/condition, given as a discrete number and unit of measurement
☒	☐	A statement on whether measurements were taken from distinct samples or whether the same sample was measured repeatedly
☐	☒	The statistical test(s) used AND whether they are one- or two-sided<br>*Only common tests should be described solely by name; describe more complex techniques in the Methods section.*
☒	☐	A description of all covariates tested
☒	☐	A description of any assumptions or corrections, such as tests of normality and adjustment for multiple comparisons
☐	☒	A full description of the statistical parameters including central tendency (e.g. means) or other basic estimates (e.g. regression coefficient) AND variation (e.g. standard deviation) or associated estimates of uncertainty (e.g. confidence intervals)
☐	☒	For null hypothesis testing, the test statistic (e.g. *F*, *t*, *r*) with confidence intervals, effect sizes, degrees of freedom and *P* value noted<br>*Give P values as exact values whenever suitable.*
☒	☐	For Bayesian analysis, information on the choice of priors and Markov chain Monte Carlo settings
☒	☐	For hierarchical and complex designs, identification of the appropriate level for tests and full reporting of outcomes
☒	☐	Estimates of effect sizes (e.g. Cohen's *d*, Pearson's *r*), indicating how they were calculated

Our web collection on statistics for biologists contains articles on many of the points above.

Software and code

Policy information about availability of computer code

Data collection	Sequencing was carried out in a BGISEQ platform using PE100 reads
Data analysis	Clean reads were aligned with HISAT. Data analysis was carried out by BGI.

For manuscripts utilizing custom algorithms or software that are central to the research but not yet described in published literature, software must be made available to editors and reviewers. We strongly encourage code deposition in a community repository (e.g. GitHub). See the Nature Portfolio guidelines for submitting code & software for further information.

Data

Policy information about availability of data

All manuscripts must include a data availability statement. This statement should provide the following information, where applicable:

- Accession codes, unique identifiers, or web links for publicly available datasets
- A description of any restrictions on data availability
- For clinical datasets or third party data, please ensure that the statement adheres to our policy

RNA-seq raw data was deposited in the Short Read Archive (SRA) under the study reference PRJNA666323

Research involving human participants, their data, or biological material

Policy information about studies with human participants or human data. See also policy information about sex, gender (identity/presentation), and sexual orientation and race, ethnicity and racism.

Reporting on sex and gender	N/A
Reporting on race, ethnicity, or other socially relevant groupings	N/A
Population characteristics	N/A
Recruitment	N/A
Ethics oversight	N/A

Note that full information on the approval of the study protocol must also be provided in the manuscript.

Field-specific reporting

Please select the one below that is the best fit for your research. If you are not sure, read the appropriate sections before making your selection.

☒ Life sciences ☐ Behavioural & social sciences ☐ Ecological, evolutionary & environmental sciences

For a reference copy of the document with all sections, see nature.com/documents/nr-reporting-summary-flat.pdf

Life sciences study design

All studies must disclose on these points even when the disclosure is negative.

Sample size	No statistical approach was used to predetermine sample size. Sample size is specified in Fig.3 panels. For Figures 4-6 sample size was always five biological replicates (N=5). For the RNA-seq, we analyzed all the genotypes and treatments in triplicate (n = 3) except for gh3oct which could only be analyzed in duplicate (N=2) since one replicate from each treatment failed while preparing the libraries. Sample sizes were determined based on similar experiments on previous experiments.
Data exclusions	N/A
Replication	N/A
Randomization	N/A
Blinding	N/A

Reporting for specific materials, systems and methods

We require information from authors about some types of materials, experimental systems and methods used in many studies. Here, indicate whether each material, system or method listed is relevant to your study. If you are not sure if a list item applies to your research, read the appropriate section before selecting a response.

Materials & experimental systems

n/a	Involved in the study	
☒	☐	Antibodies
☒	☐	Eukaryotic cell lines
☒	☐	Palaeontology and archaeology
☒	☐	Animals and other organisms
☒	☐	Clinical data
☒	☐	Dual use research of concern
☒	☐	Plants

Methods

n/a	Involved in the study	
☒	☐	ChIP-seq
☒	☐	Flow cytometry
☒	☐	MRI-based neuroimaging

Plants

Seed stocks	The dao1-1 (N593162, Porco et al. 2016), dao2-1 (N694298, Zhang et al 2016), gh3oct (Casanova-Saez et al 2022), ugt74d1 (N859668, Tanaka et al. 2014), ugt84b1 (Mateo-Bonmatí et al 2021), and ilr1-1 iar3-2 ill2-1 (Rampey et al. 2004) were previously described.
Novel plant genotypes	Iin this report we have generated three CRISPR/Cas9 constructs targeting, DAO1 and DAO2 (#1), DAO1 (#2) and DAO1 (#3). Transforming #1 into Col-0, ugt74d1 and gh3oct, we obtained new allele of DAO2, ugt74d1 dao1-5 dao2-6, and gh3oct dao2-7. Transforming #2 into dao2-1, we obtained dao1-4 dao2-1. Transforming gh3 oct with #3, we obtained gh3 oct dao1-6.
Authentication	N/A
